# Zebrafish *pten* Genes Play Relevant but Distinct Roles in Antiviral Immunity

**DOI:** 10.3390/vaccines8020199

**Published:** 2020-04-26

**Authors:** Patricia Pereiro, Antonio Figueras, Beatriz Novoa

**Affiliations:** Instituto de Investigaciones Marinas (IIM), Consejo Superior de Investigaciones Científicas (CSIC), C/ Eduardo Cabello 6, 36208 Vigo, Spain; patriciapereiro@iim.csic.es (P.P.); antoniofigueras@iim.csic.es (A.F.)

**Keywords:** zebrafish, PTEN, phosphatidylinositol 3–kinase (PI3K)/AKT, immune response, SVCV, antiviral

## Abstract

The PTEN (phosphatase and TENsin homolog on chromosome 10) gene encodes a bifunctional phosphatase that acts as a tumor suppressor. However, PTEN has been implicated in different immune processes, including autophagy, inflammation, regulation of natural killer (NK) cell cytolytic activity and type I interferon responses. Unlike mammals, zebrafish possess two *pten* genes (*ptena* and *ptenb*). This study explores the involvement of both zebrafish *pten* genes in antiviral defense. Although *ptena*^−/−^ and *ptenb*^−/−^ larvae were more susceptible to Spring viremia of carp virus (SVCV), the viral replication rate was lower in the mutant larvae than in the wild-type larvae. We observed that both mutant lines showed alterations in the transcription of numerous genes, including those related to the type I interferon (IFN) system, cytolytic activity, autophagy and inflammation, and some of these genes were regulated in opposite ways depending on which *pten* gene was mutated. Even though the lower replication rate of SVCV could be associated with impaired autophagy in the mutant lines, the higher mortality observed in the *ptena*^−/−^ and *ptenb*^−/−^ larvae does not seem to be associated with an uncontrolled inflammatory response.

## 1. Introduction

PTEN (phosphatase and TENsin homolog on chromosome 10) is a tumor suppression gene that is mutated in a wide variety of tumors [1]. This protein acts as a bifunctional phosphatase of lipids and proteins [2]. As a negative regulator of the phosphatidylinositol 3–kinase (PI3K)/AKT pathway due to its PI (phosphoinositide) 3-phosphatase activity, PTEN is involved in several cellular mechanisms, including proliferation, cell migration, apoptosis, cell survival, and metabolism [1]. Nevertheless, the recognized activities of PTEN have been expanded to include other phosphatase-independent activities and even PI3K/AKT pathway-independent activities [3]. An example of this type of activity is the role of PTEN in the maintenance of chromosome integrity and regulation of DNA damage repair [4,5,6].

Some publications have reported the involvement of mammalian PTEN in different immunological processes, including the activity, proliferation, survival, and differentiation of lymphocytes [7,8,9,10]. The PI3K/AKT pathway also has the ability to limit the activation of certain inflammatory mechanisms [11,12,13]; therefore, PTEN acts as a pro-inflammatory factor. Most of the immune mechanisms mediated by PI3K/AKT involve the mammalian target of rapamycin (mTOR) [14]. Activation of the PI3K/AKT pathway leads to the phosphorylation and subsequent activation of mTOR. Although mTOR is a central integrator of cellular metabolism, its activity affects numerous aspects of immunity [14,15]. Indeed, mTOR is an inhibitor of the autophagic process [16,17], which is a pivotal mechanism in metabolic regulation but can also directly impact the immune response and pathogen clearance [18].

Recently, PTEN has been described as a negative regulator of natural killer (NK) cell cytolytic activities [19] and as an inducer of the type I interferon (IFN) system in humans [20]. Overexpression of PTEN results in diminished NK cell cytolytic activity, whereas loss of PTEN increases NK cell cytotoxic properties [19]. This activity of PTEN is mediated via the activation of the PI3K/AKT and mitogen-activated protein kinase (MAPK) pathways and affects the organization of the immunological synapse components and the consequent mobilization of cytolytic mediators towards the target cell [19]. On the other hand, the activation of the type I IFN system is independent of the PI3K/AKT pathway and is mediated by the dephosphorylation of interferon-regulatory factor 3 (IRF3) by PTEN; the dephosphorylation of IRF3 is essential for its activation and import into the nucleus [20].

Unlike in mammals, two *pten* genes (*ptena* and *ptenb*) have been identified in zebrafish (*Danio rerio*) [21]. Zebrafish lines harboring mutant *ptena* (*ptena*^hu1864^) and *ptenb* (*ptenb*^hu1435^) alleles, which contain nonsense mutations in exons 2 and 3, respectively, were established by Faucherre et al. [22]. These mutations are positioned upstream of the phosphatase catalytic site, and no functional proteins are produced [22]. Although *ptena* and *ptenb* single mutant zebrafish are viable, the homozygous double mutation is embryonically lethal, and the larvae die approximately 5–6 days postfertilization; this observation shows that both genes possess at least some overlapping essential functions [22]. The homozygous deletion of Pten in mice is also lethal at embryonic stages [23]. In zebrafish, *pten* mutants have been used to study the role of *ptena* and *ptenb* in different processes, such as tumorigenesis [22,24,25], embryogenesis [21], angiogenesis [26], and hematopoiesis [27,28], among other processes. However, to the best of our knowledge, no previous research has investigated the direct effect of *pten* mutations on viral replication. Moreover, the role of the *pten* genes in the immunity of zebrafish, and fish in general, remains practically unexplored.

In this work, we analyzed the role of the zebrafish *pten* genes in the innate immune system at the organism level, and we specifically focused on the response against the rhabdovirus Spring viremia of carp virus (SVCV). We observed that the *pten* gene expression in zebrafish larvae can be altered by viral infection, and the absence of these genes affected the survival and viral replication after the SVCV challenge. Moreover, the expression of type I IFN-related genes, cytolytic granule components, pro-inflammatory genes, and autophagy-related molecules was analyzed in wild-type (WT), *ptena*^−/−^, and *ptenb*^−/−^ larvae, and the results revealed interesting evidence about the differential roles of the *pten* genes in immunity and response against viral infections. The rescue of the *pten* genes clearly confirmed their involvement in the expression of numerous immune genes and their role in survival after the SVCV challenge.

## 2. Materials and Methods

### 2.1. Zebrafish, Virus, and ZF4 Cell Line

Wild-type (WT), *ptena*^−/−^, and *ptenb*^−/−^ zebrafish larvae were obtained from our experimental facility, where zebrafish are maintained following established protocols [29,30]. Fish care and challenge experiments were conducted according to the guidelines of the Consejo Superior de Investigaciones Científicas (CSIC) National Committee on Bioethics under approval number ES360570202001/17/FUN.01/INM06/BNG.

The rhabdovirus Spring viremia of carp virus (SVCV, isolate 56/70) was propagated in epithelioma papulosum of cyprinid (EPC) cells (ATCC CRL-2872) and titrated in 96-well plates. The 50% tissue culture infectious dose (TCID_50_)/mL was calculated according to the Reed and Muench method [31].

The fibroblastic-like cell line ZF4, which was derived from 1-day-old zebrafish embryos (ATCC CRL-2050) [32], was cultured in Dulbecco’s modified Eagle’s medium (DMEM/F12; Gibco, Carlsbad, CA, USA) supplemented with 100 mg/mL Primocin (InvivoGen, San Diego, CA, USA) and 10% fetal bovine serum (FBS) at 26 °C.

### 2.2. Experimental Infections of Zebrafish Larvae

As in other cyprinids, SVCV has been shown to cause severe disease in zebrafish [33]. Different infection models with SVCV were applied to zebrafish larvae, and important mortalities were reached both by microinjection into the duct of Cuvier [34,35,36] and by bath challenge [37,38]. For this, we used both infection routes to analyze the mortality caused by the virus in WT, *ptena*^−/−^, and *ptenb*^−/−^ larvae.

Bath infections with SVCV were conducted in larvae at 4 days post-fertilization (dpf) using 6-well plates with 10 larvae/well and 3 replicates per zebrafish line (WT, *ptena*^−/−^, and *ptenb*^−/−^). The larvae were maintained in a volume of 6 mL of water with a SVCV concentration of 6.8 × 10^4^ TCID_50_/mL. Every day, a volume of 2 mL was replaced with new water. Mortality was recorded over the next 5 days postinfection (dpi). To confirm the results, this experiment was repeated three times. To analyze the viral replication in the different zebrafish lines, samples were also harvested at 24 and 48 h postinfection (3 biological replicates, 10 larvae/replicate). The samples were stored at −80 °C until RNA isolation.

For the microinjection experiments, 3 dpf larvae received 2 nL of an SVCV suspension (5 × 10^4^ TCID_50_/mL) or the same volume of phosphate-buffered saline (PBS) in the duct of Cuvier by microinjection with a glass microneedle coupled to a Narishige MN-151 micromanipulator and using a Narishige IM-30 microinjector. Three biological replicates for each line, each including 10 larvae, were maintained in 6-well plates in a volume of 6 mL, and the mortality was assessed over the next 5 dpi. This experiment was repeated three times. In addition to the mortality experiment, a total of 50 larvae from each line were infected with the SVCV, and 50 were inoculated with PBS. After 24 h, samples were harvested (5 biological replicates, 10 larvae/replicate) to analyze the viral replication (in two different microinjection experiments to confirm the results), the expression levels of both *ptena* and *ptenb* genes, and the transcription of numerous immune-related genes. The samples were stored at −80 °C until RNA isolation.

### 2.3. Expression Plasmids

The zebrafish *ptena* and *ptenb* genes were amplified by PCR (primers in Table S1 in Supplementary Material), and the PCR products were cloned using a pcDNA 3.1/V5-His TOPO TA Expression Kit (Invitrogen, Waltham, MA, USA), but the V5 epitope and the polyhistidine (6xHis) tag were not included. One Shot TOP10F’ competent *E. coli* (Invitrogen) was transformed to generate the constructs (pcDNA3.1-*ptena* and pcDNA3.1-*ptenb*). The plasmids were purified using a PureLink HiPure Plasmid Midiprep Kit (Invitrogen).

To assess the correct replication of the plasmids, ZF4 cells (1010 cells/treatment) were transfected with 45 µg of the *ptena* (pMCV1.4-*ptena*) or *ptenb* (pMCV1.4-*ptenb*) expression plasmid and the empty plasmid (pMCV1.4) using the Neon Transfection System (Invitrogen) (settings: 1400 V, 20 ms, and one electric pulse). Each group of electroporated cells was resuspended in 1.5 mL of DMEM/F12 + 10% FBS and seeded in 24-well plates (500 µL/well; 3 replicates per treatment). After 48 h, the cells were collected and stored at −80 °C until RNA isolation.

### 2.4. Rescue of ptena and ptenb Expression

The pcDNA3.1-*ptena* and pcDNA3.1-*ptenb* plasmids and the corresponding control empty plasmid (pcDNA3.1) were microinjected into zebrafish embryos at the one-cell stage (*ptena*^−/−^ and *ptenb*^−/−^) at a concentration of 150 pg/egg (final volume of 2 nL, diluted in PBS). WT embryos were also inoculated with the same concentration and volume of pcDNA3.1. Four days after the plasmid injection (4 dpf larvae), a bath infection with the SVCV was conducted as described above. This experiment was conducted three times. Additionally, samples were also harvested from uninfected larvae to analyze the expression of some genes that were differentially regulated in the *ptena*^−/−^ or *ptenb*^−/−^ larvae compared to that in the WT larvae (5 biological replicates, 5–6 larvae/replicate).

### 2.5. RNA Isolation, cDNA Synthesis, and Gene Expression Analysis

Total RNA isolation was performed using a Maxwell 16 LEV Simply RNA Tissue Kit (Promega, Madison, WI, USA) following the manufacturer’s guidelines. cDNA synthesis was conducted with the NZY First-Strand cDNA Synthesis kit (NZYTech, Lisbon, Portugal). The specific qPCR primers were designed using the Primer3 program [39], and their amplification efficiency was calculated by analyzing seven serial, two-fold dilutions of cDNA from unstimulated zebrafish with the threshold cycle (CT) slope method [40]. The primer sequences used in this work are listed in Table S1. The individual qPCR reactions were carried out in a 25 µL reaction volume using 12.5 µL of SYBR Green PCR Master Mix (Applied Biosystems, Foster City, CA, USA), 10.5 µL of ultrapure water (Sigma-Aldrich, St. Louis, MO, USA), 0.5 µL of each specific primer (10 µM), and 1 µL of two-fold diluted cDNA template in MicroAmp optical 96-well reaction plates (Applied Biosystems). All the reactions were performed using technical triplicates in a 7300 Real-Time PCR System Thermocycler (Applied Biosystems) with an initial denaturation step (95 °C, 10 min) that was followed by 40 cycles of a denaturation step (95 °C, 15 s) and one hybridization-elongation step (60 °C, 1 min). The relative expression of each gene was normalized using 18S ribosomal RNA as a reference gene and calculated using the Pfaffl method [40].

### 2.6. Western Blot

Zebrafish larvae pools (25 larvae/pool) were collected at 4 dpf and homogenized in 500 µL of lysis buffer (25 mM 4-(2-hydroxyethyl)-1-piperazineethanesulfonic acid (HEPES, Gibco, Carlsbad, CA, USA), 5 mM ethylene glycol tetraacetic acid (EGTA, Sigma-Aldrich, St. Louis, MO, USA), 5 mM dithiothreitol (DTT, Thermo Fisher, Waltham, MA, USA), 1% protease inhibitor cocktail (Sigma-Aldrich, St. Louis, MO, USA), and 10% phosphatase inhibitor cocktail (Thermo Fisher Waltham, MA, USA) in PBS (Gibco, Carlsbad, CA, USA)). The samples were maintained on ice during the whole procedure to avoid protein denaturation. The homogenates were centrifuged at 10,000× *g* for 10 min at 4 °C, and the resulting supernatants were recovered. The protein suspensions were mixed with 1× Laemmli sample buffer (Bio-Rad, Hercules, CA, USA), resolved in a 4%–20% Mini-PROTEAN TGX gel (Bio-Rad, Hercules, CA, USA) and transferred to a polyvinylidene difluoride (PVDF) membrane (Bio-Rad, Hercules, CA, USA). The membrane was blocked for 2 h with 3% (w/v) bovine serum albumin (BSA) in tris buffered saline with tween 20 (TBST) buffer (20 mM Tris, 0.5 M NaCl, and 0.1% Tween 20) and incubated for 1 h at room temperature with the corresponding primary antibodies diluted in 1% BSA-TBST buffer: rabbit anti-LC3A/B (Cell Signaling Technology, Danvers, MA, USA, #4108; dilution 1:500); rabbit anti-phospho-S6 ribosomal protein (Cell Signaling Technology, Danvers, MA, USA, #2215; dilution 1:300); or rabbit anti-phospho-4E-BP1 (Cell Signaling, #2855; dilution 1:200). After washing, the membrane was incubated with a goat anti-rabbit immunoglobulin G (IgG) with horseradish peroxidase (HRP) secondary antibody (Sigma-Aldrich, St. Louis, MO, USA, #A6154; dilution 1:6000), and signals were detected by chemiluminescence with Luminata™ Forte Western HRP substrate (Millipore, Burlington, MA, USA). A mouse monoclonal anti-actin antibody (Chemicon, Temecula, CA, USA, #MAB1501; dilution 1:5000) was used as a control and detected with the goat anti-mouse IgG-HRP secondary antibody (Sigma-Aldrich, St. Louis, MO, USA, #A4416; dilution 1:6000). The bands were visualized and analyzed with a ChemiDoc XRS Plus system (Bio-Rad, Hercules, CA, USA).

### 2.7. Measurement of Caspase a (Caspa) Activity 

A total of 50 zebrafish larvae (4 dpf) from WT, *ptena*^−/−^, and *ptenb*^−/−^ lines were infected by bath with SVCV (6.8 × 10^4^ TCID_50_/mL) as described above, and another 50 larvae served as uninfected controls. After 24 h, 5 pools of 10 larvae per pool were collected and homogenized in 25 mM HEPES, 5 mM EGTA, 5 mM DTT, protease and phosphatase inhibitor cocktail in PBS. The activity of caspase a (equivalent to caspase 1 in mammals) was measured with a Caspase-Glo 1 Inflammasome Assay (Promega) following the manufacturer’s instructions. The experiment was repeated twice, with 5 biological replicates and two technical replicates.

### 2.8. Statistical Analysis

Survival curves and gene expression results are represented graphically as the mean ± standard error (SE) of the biological replicates, and the graphs were generated using GraphPad Prism 7 (GraphPad Software Inc., San Diego, CA, USA). The Kaplan–Meier survival curves were analyzed with a log-rank (Mantel–Cox) test. The expression data were analyzed using the Mann–Whitney U test. Significant differences are displayed as ***/^###^ (0.0001 < *p* < 0.001), **/^##^ (0.001 < *p* < 0.01) or */^#^ (0.01 < *p* < 0.05) or with different letters (a, b).

## 3. Results

### 3.1. Expression of the ptena and ptenb Genes in Control Larvae and after SVCV Infection

**Figure 1:**
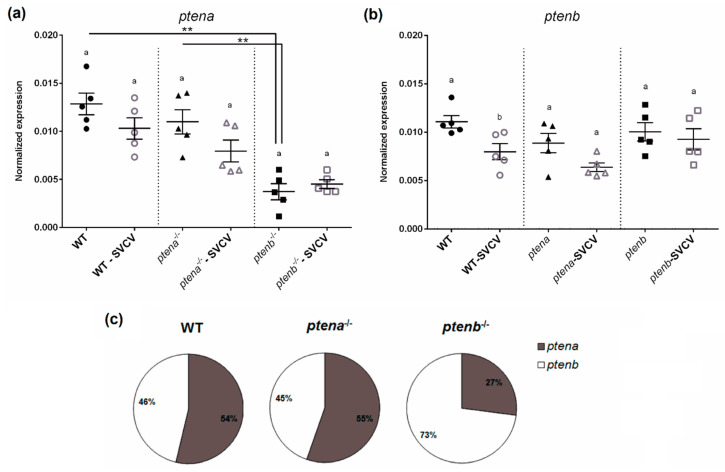
Gene expression of *ptena* and *ptenb* in zebrafish larvae. The expression of the *ptena* (**a**) and *ptenb* (**b**) genes in healthy wild-type, *ptena*^−/−^, and *ptenb*^−/−^ zebrafish larvae and at 24 h after a Spring viremia of carp virus (SVCV) challenge. The expression of the different genes was normalized to the expression of the *18S ribosomal RNA* gene. The graphs represent the means ± standard error means (SEMs) of 5 biological replicates. Statistically significant differences between uninfected and SVCV-infected individuals from the same zebrafish line are represented with different letters, whereas differences among the zebrafish lines are represented with asterisks as follows: ** (0.001 < *p* < 0.01. (**c**) mRNA proportion of the *pten* genes in the healthy larvae from the three zebrafish lines.

The expression of *ptena* and *ptenb* was analyzed in the absence and presence of SVCV infection in WT, *ptena*^−/−^, and *ptenb*^−/−^ larvae. Although no mutant line produced the corresponding functional protein, we wanted to determine if the absence of each functional protein is compensated with a higher expression of the other form of *pten* or if the organism increases the transcription of the mutated form. Interestingly, under naïve conditions, the *ptenb*^−/−^ larvae showed lower transcription of the *ptena* gene compared to the WT and *ptena*^−/−^ larvae (Figure 1a); however, the *ptena*^−/−^ larvae did not show significant differences in the expression of *ptenb* compared to the WT and *ptenb*^−/−^ larvae (Figure 1b). Therefore, in the case of *ptenb*^−/−^, it seems that the absence of the functional Ptenb protein also generated a deficiency in the level of Ptena. Moreover, the mutations did not increase the transcription of the corresponding mutated gene in any case.

Compared to the uninfected controls, the expression of *ptena* and *ptenb* showed a tendency towards downregulation 24 h after the SVCV challenge in the WT and *ptena*^−/−^ larvae (Figure 1a,b), although only the difference in the expression of *ptenb* in the WT larvae was found to be statistically significant (Figure 1b). The proportional transcription of *ptena* and *ptenb* under naïve conditions showed a very similar ratio in the WT and *ptena*^−/−^ larvae, with a slightly higher transcription of *ptena*; however, in the case of the *ptenb*^−/−^ larvae, a much higher proportion of *ptenb* mRNA was observed (Figure 1c), which is due to the lower expression of *ptena* in this mutant line (Figure 1a). 

### 3.2. ptena and ptenb Deficiency Reduces Survival after SVCV Challenge

**Figure 2:**
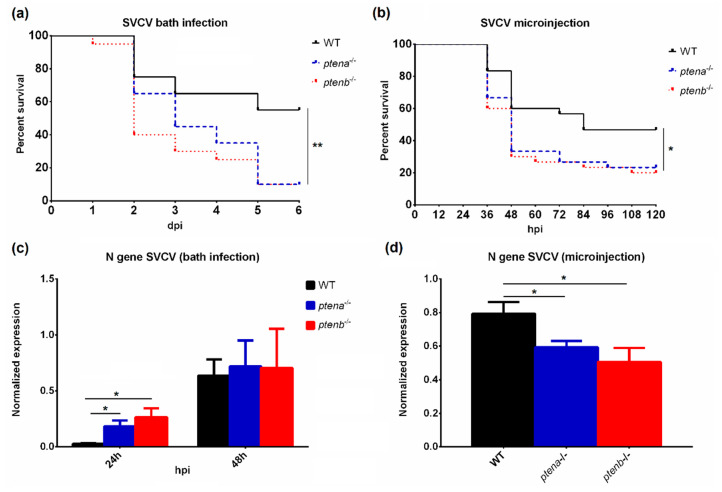
Effects of SVCV infection in wild-type and *pten*-mutant zebrafish larvae. Kaplan–Meier survival curves of the different zebrafish lines after SVCV challenge by bath infection (**a**) or microinjection into the duct of Cuvier (**b**). The statistically significant differences were determined with a log-rank (Mantel–Cox) test. The replication of the SVCV was analyzed by qPCR of the N gene at 24 h and 48 h after the bath challenge (**c**) and 24 h after the microinjection infection (**d**). The graphs represent the means ± SEMs of 3 and 5 biological replicates, respectively. In all the graphs, significant differences are displayed as ** (0.001 < *p* < 0.01) or * (0.01 < *p* < 0.05).

To analyze the susceptibility of the WT, *ptena*^−/−^, and *ptenb*^−/−^ zebrafish larvae to SVCV, two different infection protocols were performed. In the bath challenge experiment, the *ptena* and *ptenb* mutant larvae showed a significantly lower survival rate than the WT larvae (10% for both mutants and 55% for WT) (Figure 2a). On the other hand, the microinjection of the virus into the duct of Cuvier also led to significant differences in the survival rate (23.3% and 20% for *ptena*^−/−^ and *ptenb*^−/−^, respectively, and 46.67% for WT) (Figure 2b).

### 3.3. SVCV Proliferation in WT and ptena and ptenb Mutant Zebrafish

Differences in SVCV replication rates were analyzed in both infection experiments by detecting the transcription of the SVCV nucleoprotein (N) gene. Interestingly, during the bath infection experiment, the viral replication rate was higher in the mutant fish (in both *ptena*^−/−^ and *ptenb*^−/−^) than in the WT fish at 24 hpi, but after 48 h, these differences among the zebrafish lines disappeared, and comparable SVCV replication was observed (Figure 2c). In contrast, the microinjection experiment showed a significantly lower SVCV replication rate in the mutant lines than in the WT line (Figure 2d).

### 3.4. Effect of the ptena and ptenb Mutations on mTOR Activity

**Figure 3:**
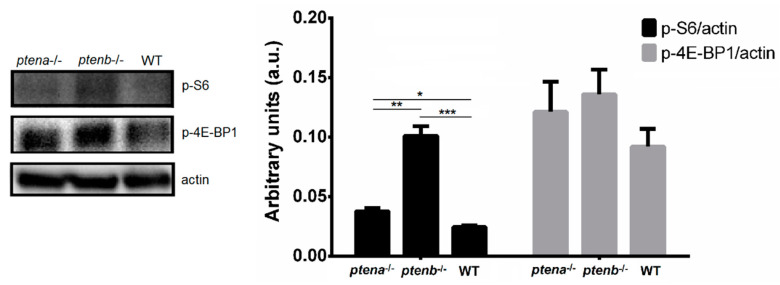
Western blot analysis of the phosphorylation of the mammalian target of rapamycin (mTOR) downstream proteins S6 (p-S6) and 4E-BP1 (p-4E-BP1). The intensity of the bands was normalized to the actin protein level. The graphs represent the means ± SEMs of 5 pools of larvae. Statistically significant differences are displayed as *** (0.0001 < *p* < 0.001), ** (0.001 < *p* < 0.01) or * (0.01 < *p* < 0.05).

The phosphorylation of the S6 ribosomal protein and 4E-BP1 is widely used to measure mTOR activation. In mammals, Pten mutation leads to mTOR hyperactivation. To analyze the involvement of both zebrafish *pten* genes in the activity of mTOR, we conducted Western blot analysis of the phosphorylated form of both mTOR-downstream proteins. As expected, the levels of phosphorylated S6 and 4E-BP1 were higher in both mutant lines, especially in the *ptenb*^−/−^ line, but only the differences in p-S6 were statistically significant (Figure 3). This observation shows that, as in mammals, the mutation of the *pten* genes increases the activation of mTOR.

### 3.5. pten Mutations Alter the Immune Profile of Zebrafish Larvae

#### 3.5.1. Type I IFN Axis

**Figure 4:**
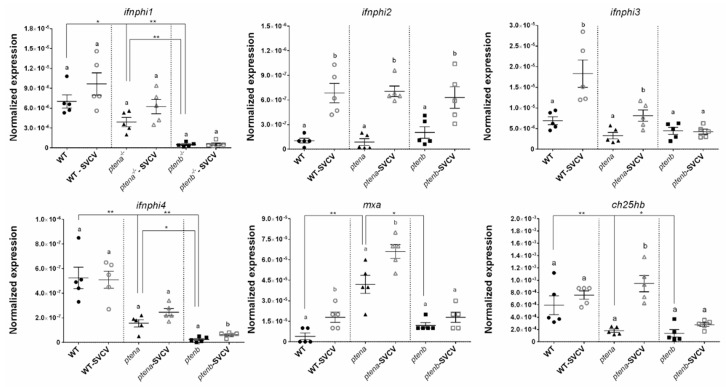
Expression of type I interferon (IFN)-related genes in wild-type (WT), *ptena*^−/−^, and *ptenb*^−/−^ zebrafish larvae under healthy and SVCV-infected conditions. The expression of the different genes was normalized to the expression of the *18S ribosomal RNA* gene. The graphs represent the means ± SEMs of 5 biological replicates. Statistically significant differences between the uninfected and SVCV-infected individuals from the same zebrafish line are represented with different letters, whereas differences among the zebrafish lines are represented with asterisks as follows: ** (0.001 < *p* < 0.01) or * (0.01 < *p* < 0.05).

The constitutive expression of the four zebrafish *ifnphi* genes was analyzed in the WT, *ptena*^−/−^, and *ptenb*^−/−^ larvae. The expression of *ifnphi1* and *ifnphi4* was significantly higher in the WT line than in both mutant lines and higher in the *ptena*^−/−^ line than in the *ptenb*^−/−^ line (Figure 4). Nevertheless, neither *ifnphi2* nor *ifnphi3* showed statistically significant differences among the larval groups (Figure 4). Although *ifnphi1* and *ifnphi4* were not affected by the SVCV challenge in the WT and *ptena*^−/−^ larvae, *ifnphi4* was slightly but significantly overexpressed 24 h postchallenge only in the *ptenb*^−/−^ larvae (Figure 4). On the other hand, *ifnphi2* was overexpressed after infection in all the larvae groups, and *ifnphi3* was overexpressed in the WT and *ptena*^−/−^ larvae.

When one of the multiple interferon-stimulated genes (ISGs), *myxovirus resistance gene a* (*mxa*), was analyzed, a higher expression of this gene was found in the *ptena*^−/−^ larvae than in the other groups (Figure 4). This result was unexpected because none of the *ifnphi* genes was highly expressed in these mutant fish compared to the WT and *ptenb*^−/−^ zebrafish. Significant upregulation of this gene was observed after SVCV challenge in the WT and *ptena*^−/−^ larvae (Figure 4). Although cholesterol-25-hydroxylase genes have been described as ISGs, a previous report showed that the association of these genes, particularly that of the main *ch25h* gene induced after SVCV challenge, *ch25hb*, may not be completely conserved in zebrafish. However, the antiviral function of this gene has been established [41]. The expression of *ch25hb* was significantly lower in the *ptena*^−/−^ and *ptenb*^−/−^ larvae than in the WT larvae in the absence of infection (Figure 4). However, after SVCV challenge, although the three zebrafish lines tended to increase the expression of this gene, only the *ptena*^−/−^ larvae significantly increased the expression of *ch25hb* after 24 h.

#### 3.5.2. Cytotoxic Profile

**Figure 5:**
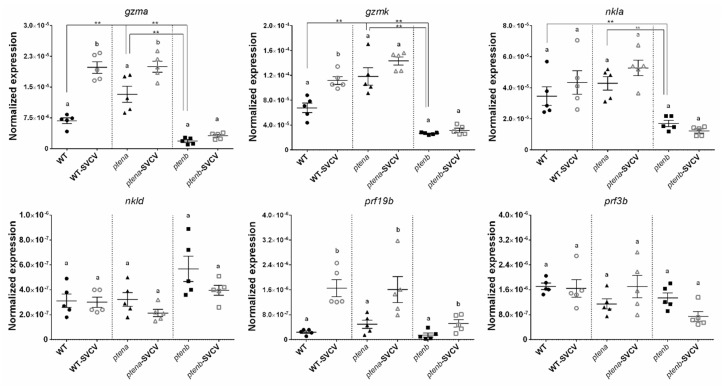
Expression of cytotoxic granule-related genes in WT, *ptena*^−/−^, and *ptenb*^−/−^ zebrafish larvae under healthy and SVCV-infected conditions. The expression of the different genes was normalized to the expression of the *18S ribosomal RNA* gene. The graphs represent the means ± SEMs of 5 biological replicates. Statistically significant differences between the uninfected and SVCV-infected individuals from the same zebrafish line are represented with different letters, whereas differences among the zebrafish lines are represented with asterisks as follows: ** (0.001 < *p* < 0.01).

The analyzed granzyme genes, *gzma* and *gzmk*, showed the highest expression in the *ptena*^−/−^ zebrafish, followed by the WT zebrafish, and the lowest expression was observed in the *ptenb*^−/−^ zebrafish. However, none of these granzymes was overexpressed in the *ptenb*^−/−^ larvae after SVCV microinjection, but significant upregulation of *gzma* and *gzmk* in the WT larvae and *gzma* in the *ptena*^−/−^ larvae was observed (Figure 5).

Although the expression of *nkla* did not differ between the WT and *ptena*^−/−^ larvae, the transcription of this gene was lower in the *ptenb*^−/−^ larvae. On the other hand, significant differences in the expression of *nkld* were not observed among the three lines, although a trend towards a higher expression in the *ptenb*^−/−^ larvae could be observed. Neither *nkla* nor *nkld* was overexpressed after viral challenge (Figure 5).

The perforin genes *prf19b* and *prf3b* were not differentially expressed among the three lines, and only prf19b was overexpressed after SVCV in all the lines (Figure 5). Therefore, *pten* mutations do not seem to affect the transcription of these perforins in larvae.

#### 3.5.3. Autophagy-Related Genes and Lc3 Abundance

**Figure 6:**
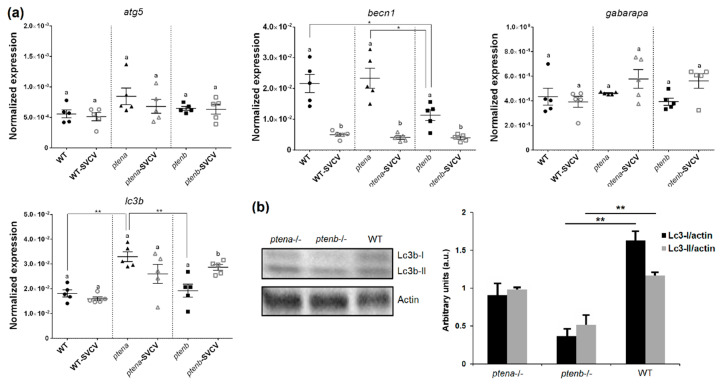
Effect of the *pten* mutations on the levels of the autophagy-related components. (**a**) Expression of the autophagy-related genes in the different zebrafish lines under healthy and SVCV-infected conditions. The expression of the different genes was normalized to the expression of the *18S ribosomal RNA* gene. The graphs represent the means ± SEMs of 5 biological replicates. Statistically significant differences between the uninfected and SVCV-infected individuals from the same zebrafish line are represented with different letters, whereas differences among the zebrafish lines are represented with asterisks as follows: ** (0.001 < *p* < 0.01) or * (0.01 < *p* < 0.05). (**b**) Western blot detection of Lc3b-I and Lc3b-II in pools of zebrafish larvae (4 dpf). The graphs represent the means ± SEMs of 5 larvae pools. Statistically significant differences between the zebrafish lines are displayed as ** (0.001 < *p* < 0.01).

The activation of mammalian target of rapamycin (mTOR) by the PI3k/AKT pathway inhibits autophagy; therefore, PTEN is an indirect inducer of autophagy [16,17]. Four genes related to this mechanism were analyzed in the zebrafish larvae: *autophagy related 5* (*atg5*), *beclin 1* (*becn1*), *GABA(A) receptor-associated protein a* (*gabarapa*), and *microtubule associated protein 1 light chain 3 beta* (*lc3b*). No significant differences were observed in *atg5* and *gabarapa* among the WT and mutant lines, and these genes were not affected by viral challenge (Figure 6a). However, *becn1* was expressed at significantly lower levels in the *ptenb*^−/−^ larvae than in the WT and *ptena*^−/−^ larvae, and this gene was downregulated in the three lines after SVCV infection (Figure 6a). Interestingly, *lc3b* had a higher basal expression in the *ptena*^−/−^ larvae than in the other lines, but after viral challenge, significant overexpression was only observed in the *ptenb*^−/−^ larvae (Figure 6a).

To analyze autophagy at the protein level, we conducted Western blot analysis of the Lc3 component. Contrary to what was observed for gene expression, total Lc3 (Lc3-I and Lc3-II) was expressed at a lower level in the *pten* mutant larvae, especially in the *ptenb*^−/−^ larvae (Figure 6b).

#### 3.5.4. Pro-Inflammatory Cytokines and Inflammasome-Related Molecules

**Figure 7:**
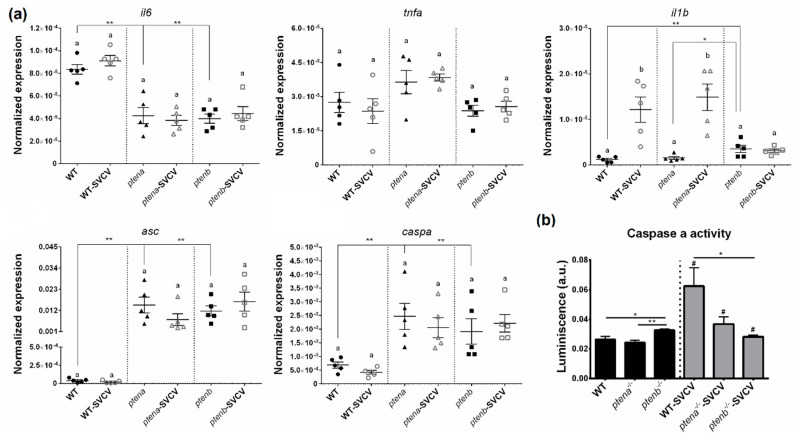
Mutations in the *pten* genes alter the inflammation-related gene expression and inflammasome activity. (**a**) Expression of pro-inflammatory cytokines and inflammasome components in the WT and *pten* mutant zebrafish larvae under healthy and SVCV-infected conditions. The expression of the different genes was normalized to the expression of the *18S ribosomal RNA* gene. The graphs represent the means ± SEMs of 5 biological replicates. Statistically significant differences between the uninfected and SVCV-infected individuals from the same zebrafish line are represented with different letters, whereas differences among the zebrafish lines are represented with asterisks as follows: ** (0.001 < *p* < 0.01) or * (0.01 < *p* < 0.05) (**b**) Measurement of caspa activity in the WT, *ptena*^−/−^, and *ptenb*^−/−^ larvae exposed or not to SVCV bath challenge for 24 h. The graphs represent the means ± SEMs of 5 biological replicates (10 larvae/replicate). Statistically significant differences between the uninfected and SVCV-infected individuals from the same zebrafish line are represented with hashes (^#^ (0.01 < *p* < 0.05)), whereas differences among the zebrafish lines are represented with asterisks (** (0.001 < *p* < 0.01) or * (0.01 < *p* < 0.05)).

To determine if the higher mortality observed in the *ptena* and *ptenb* mutant larvae was due to an exacerbated pro-inflammatory response, the levels of some of the main pro-inflammatory cytokines were analyzed. Although *tumor necrosis factor alpha* (*tnfa*) was not affected by the mutation of the *pten* genes, the transcription of *interleukin 6* (*il6*) was higher in the WT line than in the mutant lines (Figure 7a). In contrast, *interleukin 1 beta* (*il1b*) was more highly expressed in the *ptenb*^−/−^ larvae than in the WT and *ptena*^−/−^ larvae. Interestingly, after SVCV infection, *il1b* was significantly overexpressed only in the WT and *ptena*^−/−^ larvae, but it remained unaltered in the *ptenb*^−/−^ larvae, which was the zebrafish line with the highest constitutive expression of this cytokine (Figure 7a).

Since *il1b* is a pivotal member of the inflammasome, we also analyzed the expression of other members of the inflammasome complex, namely, *apoptosis-associated speck-like protein containing a caspase recruitment domain* (*asc*) and *caspase a* (*caspa*). Although *il1b* was only overexpressed in the *ptenb*^−/−^ larvae under naïve conditions, both the *asc* and *caspa* genes were more highly expressed in both *pten* mutant lines compared to the WT line. However, neither *asc* nor *caspa* showed changes in expression 24 h after SVCV infection (Figure 7a).

To assess the functionality of the inflammasome system, we further measured caspase a activity in the absence or presence of SVCV infection (Figure 7b). Under naïve conditions, the *ptenb*^−/−^ larvae showed significantly higher caspa activity that the WT and *ptena*^−/−^ larvae. Interestingly, 24 h after an SVCV bath challenge, both WT and *ptena*^−/−^ individuals significantly increased their caspa activity, especially the WT individuals, whereas a slight but significant reduction was observed in the *ptenb*^−/−^ individuals (Figure 7b).

#### 3.6. ptena and ptenb Rescue

The replication of the expression plasmids encoding *ptena* and *ptenb* was confirmed in ZF4 cells at 48 h posttransfection (Figure S1a). In addition, the correct replication of both genes was also analyzed in zebrafish larvae (4 dpf) that were microinjected with the expression plasmids at the one-cell embryo stage (Figure S1b).

**Figure 8:**
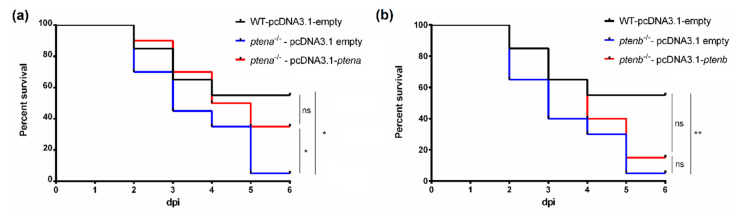
Kaplan–Meier survival curves representing the effect of the *ptena* gene rescue in *ptena*^−/−^ zebrafish embryos (**a**) and the *ptenb* gene rescue in *ptenb*^−/−^ zebrafish embryos (**b**) during bath challenge with SVCV. No mortality events were recorded for the uninfected controls. Statistically significant differences were determined with a log-rank (Mantel–Cox) test and are displayed as ** (0.001 < *p* < 0.01) or * (0.01 < *p* < 0.05); ns: non-significant differences.

**Figure 9:**
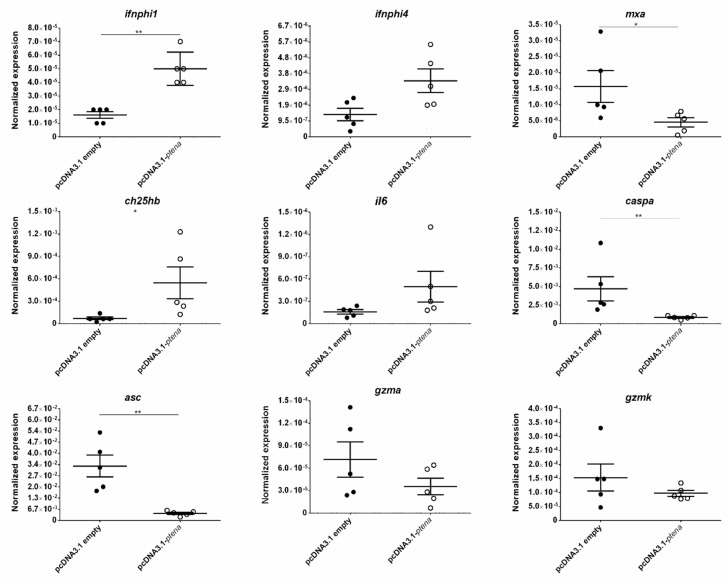
Effect of gene rescue on the expression of dysregulated genes in *ptena* mutant zebrafish larvae. The expression of the different genes was normalized to the expression of the *18S ribosomal RNA* gene. The graphs represent the means ± SEMs of 5 biological replicates. Statistically significant differences are displayed as ** (0.001 < *p* < 0.01) or * (0.01 < *p* < 0.05).

When both *pten* genes were rescued in the mutant zebrafish, we observed a significant increase in survival after SVCV challenge in the *ptena*^−/−^ fish inoculated with pcDNA3.1-*ptena* (Figure 8a) compared to the mutants inoculated with the empty plasmid. Expression analysis of some of the dysregulated genes in the *ptena*^−/−^ larvae revealed that the rescue of the *ptena* gene was able to restore their transcription (Figure 9). Generally, the genes that were expressed at lower levels in the *ptena* mutant larvae than in the WT larvae showed increased expression four days after pcDNA3.1-*ptena* injection, whereas the genes that were expressed at higher levels in the *ptena*^−/−^ larvae were downregulated after *ptena* rescue.

**Figure 10:**
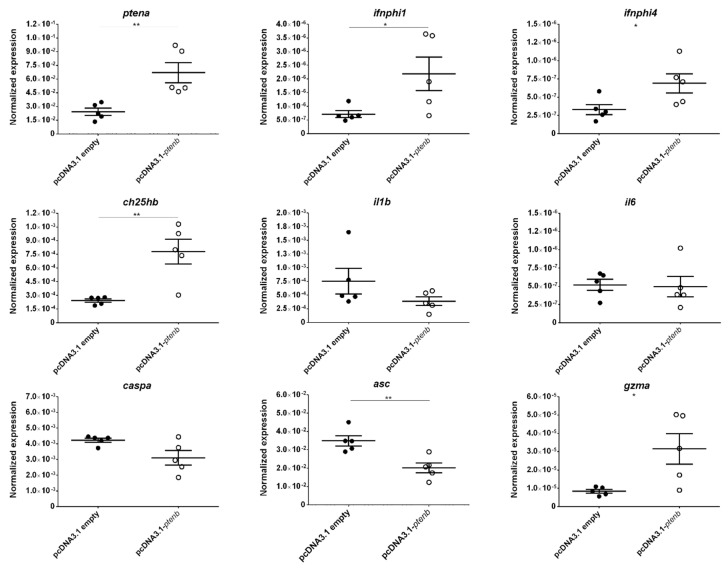
Effect of gene rescue on the expression of dysregulated genes in *ptenb* mutant zebrafish larvae. The expression of the different genes was normalized to the expression of the *18S ribosomal RNA* gene. The graphs represent the means ± SEMs of 5 biological replicates. Statistically significant differences are displayed as ** (0.001 < *p* < 0.01) or * (0.01 < *p* < 0.05).

In the case of the *ptenb*^−/−^ larvae, although no significant differences in the survival rate between the individuals inoculated with pcDNA3.1-*ptenb* or with the empty plasmid were observed at the end of the experiment, a certain delay in mortality was observed after the rescue of the *ptenb* gene (Figure 8b). As with the rescue of the *ptena* gene, after the rescue of the *ptenb* gene, most of the genes that exhibited differential expression between the *ptenb*^−/−^ and WT larvae showed altered expression that more closely resembled their expression in the WT larvae (Figure 10).

## 4. Discussion

PTEN was independently discovered by three laboratories in 1997 and is described as a tumor suppressor that is mutated in numerous cancers [42,43,44]. For that reason, investigations related to PTEN activity have mainly focused on cancer research. However, because PTEN is an inhibitor of the PI3K/AKT/mTOR pathway, it is involved in numerous cellular biological processes [1,3]. Most of the activities mediated by PTEN directly impact a variety of immune mechanisms [45].

Zebrafish is a model species that is widely used in biomedical research, including research about cancer [46,47,48]. The existence of two *pten* genes (*ptena* and *ptenb*) in zebrafish could make it difficult to use this model to study the effect of Pten mutations in cancer due to the lethality of the double homozygous mutation (*ptena*^−/−^*ptenb*^−/−^) at early developmental stages and the existence of redundant functions between both proteins [22]. Nevertheless, it has been shown that *ptenb*^−/−^ zebrafish develop ocular tumors later in life, even when *ptena* is expressed [22]. Choorapoikayil et al. [25] found that zebrafish mutants that retain a single wild-type copy of *ptena* or *ptenb* (*ptena*^+/-^*ptenb*^−/−^ or *ptena*^−/−^*ptenb*^+/-^) are viable and fertile and that those with any copy of intact *ptenb* are more susceptible to developing hemangiosarcoma than those carrying any wild-type copy of *ptena*. Based on these results, *ptenb* mutations seem to more strongly predispose individuals to tumors than *ptena* mutations [22,25]. 

Mice that are partially deficient in PTEN were more susceptible to infection with vesicular stomatitis virus (VSV) than WT mice [20]. Accordingly, the VSV titers in the livers of PTEN-mutant mice were higher than those in their WT counterparts [20]. To analyze if these mutations also increase the susceptibility to viral infection with SVCV, we evaluated the survival of WT, *ptena*^−/−^, and *ptenb*^−/−^ larvae infected with SVCV and the replication of the virus in the different lines. Surprisingly, although both mutant zebrafish lines were more susceptible to SVCV challenge than the WT line, the replication of the virus, as assessed by qPCR, was lower in the mutant lines when SVCV was administered by microinjection. When the challenge was conducted by bath, at earlier stages postinfection (24 hpi), SVCV replicated more actively in the *ptena*^−/−^ and *ptenb*^−/−^ larvae, but after 48 h, viral replication was equal among all the lines. These observations seem to indicate that although SVCV could penetrate into the mutant individuals more easily, once inside, its replication abilities would decrease when the *pten* genes were mutated. These results are not in agreement with those observed in mice that are partially deficient in PTEN, which showed higher replication of VSV compared to the WT animals [20]. However, despite the lower replication rate, the mutant zebrafish larvae were more susceptible to SVCV infection, and the survival of the mutant fish tended to be restored when the mutated gene was rescued by the microinjection of an expression plasmid. For that reason, we wanted to explore the role of both zebrafish *pten* genes in different aspects of the immune response. The involvement of *ptena* and *ptenb* in defense mechanisms has remained practically unexplored until now. Due to the main activity of PTEN as an inhibitor of the PI3K/AKT/mTOR pathway, different immune-related factors downstream of this pathway were analyzed. However, some immune regulation could also be independent of the PI3K/AKT cascade, as described for the regulation of the type I IFN system in mammals [20].

Since PTEN is an inhibitor of the PI3K/AKT/mTOR pathway, we first wanted to analyze if this function is conserved for both zebrafish genes. In mammals, PTEN mutations lead to hyperactivation of mTOR [49], which results in downstream proteins becoming phosphorylated. The phosphorylation of the S6 ribosomal protein and 4E-BP1 is usually used as a readout of mTOR activation [50,51]. The results confirmed that mTOR is especially hyperactivated in the *ptenb*^−/−^ larvae, probably because these larvae are also partially deficient in Ptena.

The type I IFN system is the main mechanism responsible for orchestrating the antiviral immune response in vertebrates. Zebrafish possess four type I IFN genes, named *ifnphi1–4* [52]. However, *ifnphi1* and *ifnphi4* (group I) and *ifnphi2* and *ifnphi3* (group II) do not interact with the same receptors [52], which shows a clear differentiation between both groups. Interestingly, the *pten* mutations only affected the constitutive expression of *ifnphi1* and *ifnphi4*, which was downregulated, especially in the *ptenb*^−/−^ larvae. This differentiation in the basal expression of group I and group II IFNs suggests differential induction pathways for the two subtypes of IFNs, and both zebrafish Pten molecules may be more involved in the synthesis of group I IFNs. Unexpectedly, when the expression of the ISG *mxa* was analyzed, higher transcription was observed in the *ptena*^−/−^ larvae, which could suggest an activation of *mxa* transcription that is independent of the type I IFN system and affected by Ptena. Contrary to that observed in mammals, the zebrafish *ch25hb* gene does not seem to be regulated by type I IFNs [41]; however, it exerts powerful antiviral activity against SVCV by increasing the synthesis of 25-hydroxycholesterol (25-HC) from cholesterol [41]. Both the *ptena*^−/−^ and *ptenb*^−/−^ larvae showed lower basal transcription of *ch25hb* than the WT larvae, although only the *ptena*^−/−^ larvae showed significantly increased transcription of this gene 24 h after SVCV microinjection. Therefore, generally, the type I IFN response is greatly altered by mutations in the *pten* genes.

Perforin/granzyme-induced apoptosis is the main pathway used by cytotoxic cells to eliminate virus-infected cells [53]. Natural killer (NK) cells and cytotoxic T lymphocytes (CTLs) contain cytolytic granules with three different components: NK-lysin, perforin, and serine-protease granzymes [53]. Although mammals usually possess only one form of Nk-lysin and perforin, zebrafish and other teleost fish possess several isoforms of these molecules [54,55]. Although the effects of PTEN on the cytotoxic activity of NK cells were attributed to the negative regulation of the release of the granule contents [19], we wanted to investigate if the gene expression of the lytic components could be affected by the *ptena* or *ptenb* mutations. Although the *ptenb*^−/−^ fish showed lower expression of some of the cytotoxic genes (*gzma*, *gzmk*, and *nkla*), the *ptena*^−/−^ fish exhibited higher expression of *gzma* and *gzmk*. Therefore, although more investigations are needed, we could assume that the *ptenb*^−/−^ and *ptena*^−/−^ larvae probably show reduced and increased cytolytic abilities, respectively. Moreover, the opposite trends in the expression of the granzyme genes in both mutant zebrafish lines could indicate the opposite regulation of these granzyme genes by both *pten* genes.

Based on the results obtained for the type I IFN system and the cytotoxic genes, at least the *ptenb*^−/−^ zebrafish seem to clearly possess an inferior antiviral response. For that reason, we would expect a higher SVCV replication rate in these animals, but contrary to that expectation, these animals showed the lowest SVCV replication rate after microinjection. In contrast, after bath challenge, SVCV showed a higher replication rate in the *ptenb*^−/−^ larvae followed by the *ptena*^−/−^ larvae at the earlier time point, which could be consistent with the gene expression results, as it was easier for the virus to penetrate into the larvae due to the impaired antiviral mechanisms. However, once inside, SVCV showed lower replication in the mutant larvae. Since mTOR is an inhibitor of autophagy, and it is known that autophagy is needed for the propagation of some viruses, including SVCV [38,56], the lower SVCV replication rate could be due to autophagy inhibition. Therefore, we analyzed the expression of pivotal genes involved in autophagy and measured the protein levels of Lc3-I and Lc3-II. However, under basal conditions, the *ptenb*^−/−^ larvae showed lower expression of becn1, and the *ptena*^−/−^ larvae showed higher expression of *lc3b*, which could indicate that both zebrafish *pten* genes regulate the expression of different autophagy components. In humans, both LC3B and BECN1 were downregulated by PTEN knockdown and upregulated by PTEN overexpression [57,58]. However, when we analyzed the abundance of Lc3-I and Lc3-II, we found that although the Lc3-II/Lc3-I ratio (an indicator of autophagy activation) was higher in both *pten* mutants, this finding occurred due to lower levels of both Lc3-I and Lc3-II, especially in the *ptenb*^−/−^ larvae. Therefore, although further investigations are needed for confirmation, the lower autophagy in the *pten* mutants could explain the lower replication rate of SVCV. Nevertheless, as mentioned above, mice partially deficient in PTEN showed a higher replication of VSV [20], which is also a rhabdovirus. This discrepancy between both viruses could be due to the pernicious effect of the autophagy in the replication of VSV [59], whereas the opposite effect is observed for SVCV [38,56]. Therefore, autophagy can benefit or reduce the viral spread depending on the virus (revised in [60]) and consequently, mutations in the PTEN genes could also provide different results for different viruses. 

However, what can explain the lower survival in response to SVCV challenge even when the virus showed a lower replication rate? mTOR activation and the consequent autophagy inhibition increase inflammasome activation [61,62,63,64]. Thus, we hypothesized that excessive inflammatory damage could be the reason for the higher susceptibility of the *pten* mutant larvae to SVCV. We analyzed the expression of different components of the inflammasome and other pro-inflammatory cytokines. Both *pten* mutants showed lower expression of *il6*, although this cytokine can act as a pro-inflammatory or anti-inflammatory molecule [65]. Nevertheless, because this cytokine is also involved in tissue repair [66,67,68] and SVCV is a highly hemorrhagic virus that generates tissue destruction in different organs [69], deficiencies in the synthesis of IL-6 could be related to the higher mortality of the *pten* mutant zebrafish through deficiencies in the repair processes. Regarding the inflammasome components, both the *ptena*^−/−^ and *ptenb*^−/−^ larvae expressed higher levels of *asc* and *caspa* than the WT larvae. Interestingly, *il1b* was only overexpressed in the *ptenb*^−/−^ larvae under basal conditions, but after viral challenge, its expression was significantly increased in the WT and *ptena*^−/−^ larvae but was not affected in the *ptenb*^−/−^ larvae. Consistent with this finding, although the caspa activity was higher in the *ptenb*^−/−^ uninfected larvae, once infected, the caspa activity was significantly inhibited in these larvae but increased in the *ptena*^−/−^ larvae, and even more so in the WT larvae. Therefore, the inflammatory status of the WT larvae after SVCV infection is higher than that in the *pten* larvae, and it is especially higher than that in the *ptenb*^−/−^. Based on this observation, we cannot conclude that the lower survival of the mutant larvae after SVCV infection is due to exacerbated inflammation. A similar response was previously observed for mutant *rag1*^−/−^ zebrafish. These animals do not possess adaptive immunity but show a higher basal innate immunity, including an increased pro-inflammatory status [70,71]; however, after SVCV challenge, the expression of some pivotal pro-inflammatory genes was lower than what was observed in the WT zebrafish [70].

This publication represents the first work providing information about the potential function of the zebrafish *pten* genes not only in the antiviral response, but also in certain cellular processes, such as autophagy. Although zebrafish possess two *pten* genes with some exclusive and even opposite effects in antiviral immunity, the information contained in this work could provide interesting clues for biomedical research, since some of the aspects explored here were not previously reported for vertebrates. Moreover, although more studies should be conducted in the future to explore additional signaling pathways, our results raise interesting questions about the potential susceptibility or resistance of the tumor cells derived from mutations in PTEN to different viruses. This could help in the development of the emergent oncolytic virus therapy, which is based in the design of recombinant viruses that can replicate specifically in tumor cells and induce cell death [72].

## 5. Conclusions

**Figure 11:**
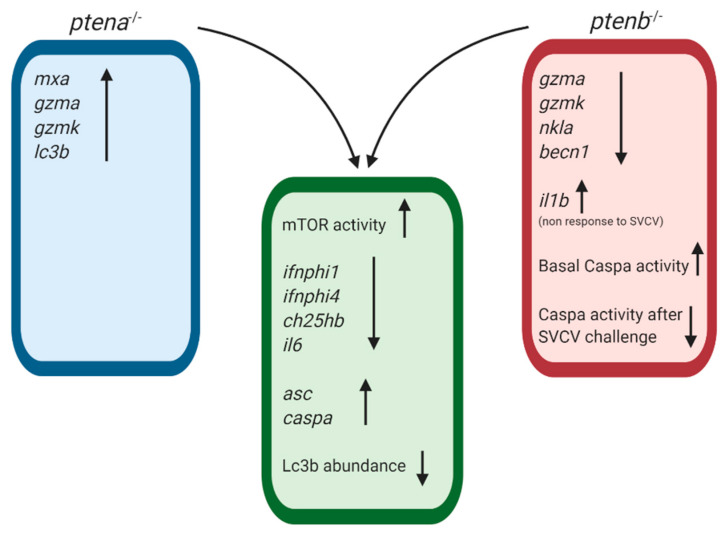
Summary graph representing the common and exclusive modulations observed in the *ptena* and *ptenb* mutant zebrafish larvae compared to WT individuals. Arrows up indicate gene overexpression or up-modulated processes compared to WT larvae; arrows down indicate gene expression inhibition or down-regulated processes compared to WT larvae.

The complexity of the PTEN regulatory functions, which affect a wide variety of biological processes, could affect survival after an immune challenge regardless of the pathogen burden and inflammatory response. More investigations will be needed to elucidate the concrete mechanisms involved in this increased susceptibility. The involvement of the *ptena* and *ptenb* genes in the immune response was clearly shown, and the rescue of these genes in the mutated larvae tended to revert both the SVCV-induced mortality and altered gene expression. Some immune genes were affected in the same way by the *ptena* and *ptenb* mutations, but interestingly, certain genes were only affected by one of these mutations, and certain genes were regulated by the mutations in opposite ways (Figure 11). These observations reflect some redundant immune functions and some specific regulatory functions of the zebrafish *pten* genes.

## Supplementary Materials

The following are available online at https://www.mdpi.com/2076-393X/8/2/199/s1, Table S1: Primer pairs used in this work, Figure S1: Replication of the expression plasmids encoding the zebrafish *ptena* and *ptenb* genes in (a) ZF4 cells transfected with the plasmids or (b) zebrafish larvae microinjected with pcDNA3.1-*ptena* or pcDNA3.1-*ptenb* at the one-cell embryo stage.
