# Supplementary material for: Zebrafish pten Genes Play Relevant but Distinct Roles in Antiviral Immunity
Source: Vaccines (Basel). 2020 Apr 26;8(2):199. doi: 10.3390/vaccines8020199 (PMC7349019; doi:10.3390/vaccines8020199)

Figure S1. Replication of the expression plasmids encoding the zebrafish ptena and ptenb genes in (a) ZF4 cells transfected with the plasmids or (b) zebrafish larvae microinjected with pcDNA3.1-*ptena* or pcDNA3.1-*ptenb* at the one-cell embryo stage. The correct replication was analysed by qPCR analysis of both pten genes. The expression of the different genes was normalized to the expression of the *18S ribosomal RNA* gene. The graphs represent the means ± SEMs of 4 and 5 biological replicates for the ZF4 cells and zebrafish larvae, respectively. Statistically significant differences with the controls are displayed as *** ($0.0001 < p < 0.001$), ** ($0.001 < p < 0.01$) or * ($0.01 < p < 0.05$).

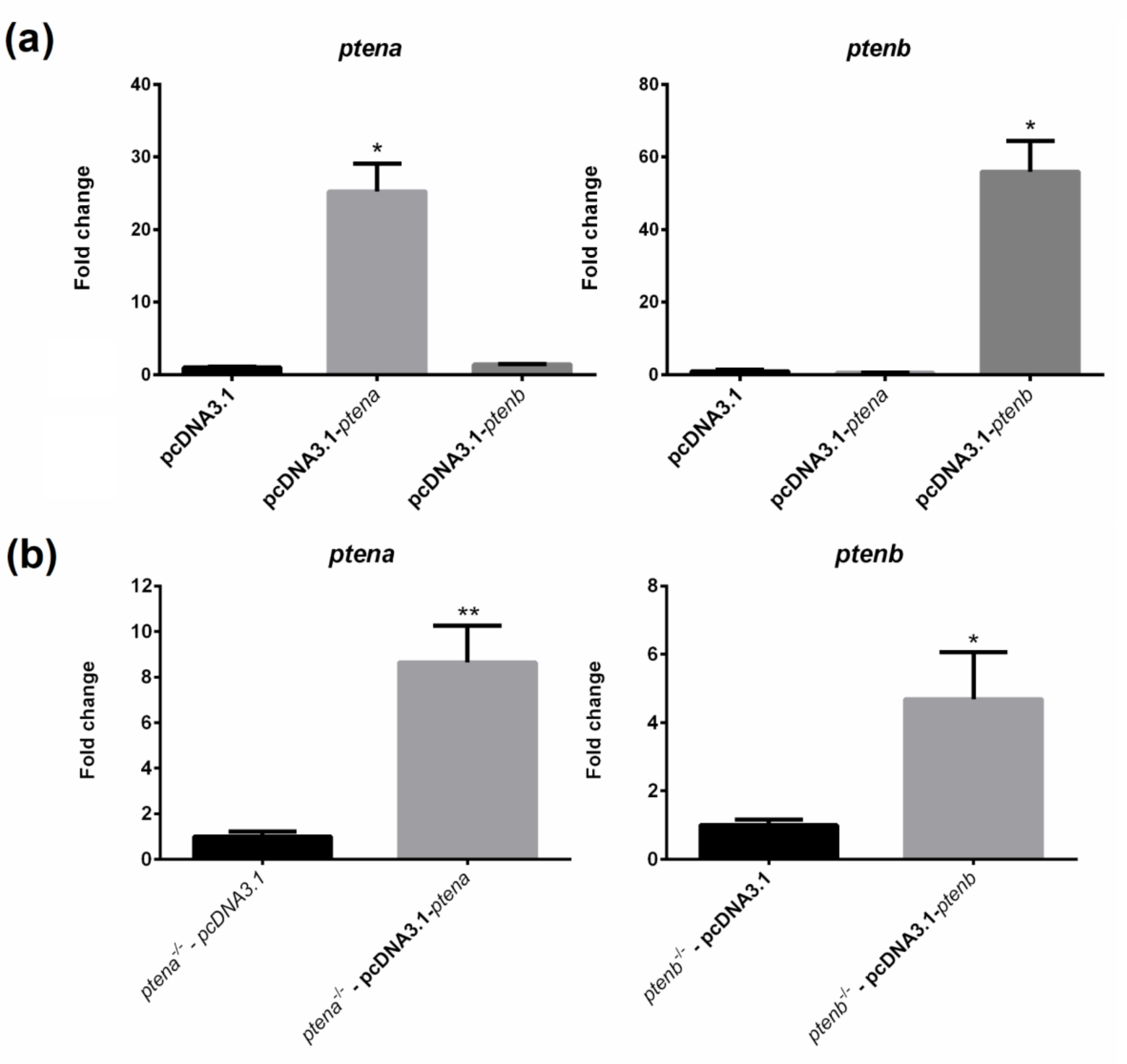

Supplement: Supplementary file 1 [file vaccines-08-00199-s001.zip › vaccines-780868-xml-sup/Figure S1.pdf]
