# Supplementary material for: Zebrafish pten Genes Play Relevant but Distinct Roles in Antiviral Immunity
Source: Vaccines (Basel). 2020 Apr 26;8(2):199. doi: 10.3390/vaccines8020199 (PMC7349019; doi:10.3390/vaccines8020199)
Supplement: Supplementary file 1 [file vaccines-08-00199-s001.zip › vaccines-780868-xml-sup/Table S1.pdf]

Supplementary Table S1. Primer pairs used in this work.

Gene	Ensembl ID	Primer sense	Sequence (5´-3')	Application
ptena	ENSDARG00000071018	Forward	GTCATGGCAATGACTGCTAAAC	ORF amplification
		Reverse	TCAGACTTTTGTAATCTGTGCGTG	
		Forward	GAGAAAGAGGAGAGCAGCGA	qPCR
		Reverse	CTCCACGGTCTTGGTGAAAT	
ptenb	ENSDARG00000056623	Forward	GCCATGGCTGCGATCATAAAGG	ORF amplification
		Reverse	TCAAACTTTAGTAATCTGTTCTTC	
		Forward	AAGCTGGAAAGGGAAGAACG	qPCR
		Reverse	ACATATCGCCTCTGACTGGG	
ifnphi1	ENSDARG00000025607	Forward	GAGCACATGAACTCGGTGAA	qPCR
		Reverse	TGCGTATCTTGCCACACATT	
ifnphi2	ENSDARG00000069012	Forward	CCTCTTTGCCAACGACAGTT	qPCR
		Reverse	CGGTTCCTTGAGCTCTCATC	
ifnphi3	ENSDARG00000070676	Forward	TTCTGCTTTGTGCAGGTTTG	qPCR
		Reverse	GGTATAGAAACGCGGTCGTC	
ifnphi4	ENSDARG00000100678	Forward	GGGGTCTCTCTGGATCTCCT	qPCR
		Reverse	ATCTGCTTCTCAGGCTCTGC	
mxa	ENSDARG00000021688	Forward	CACAGACAATCATGCCACCT	qPCR
		Reverse	TTTGCAGCTCCAAAGCAGCT	
ch25hb	ENSDARG00000045190	Forward	CGGTGAATCCCATGTTGCTT	qPCR
		Reverse	AGCTCCTCCGTAAAGTCCAAAA	
nkla	ENSDARG00000112801	Forward	GATGACGAATGACGGAGTAAAC	qPCR
		Reverse	TCTCATTCACAGCCCGGT	
nkld	ENSDARG00000044023	Forward	TGTGATCAGATCGGGTTCCT	qPCR
		Reverse	AGCACAGATGGTTCTGGCAT	
prf19b	ENSDARG00000001572	Forward	ATGGCTCTCCTTCTGTTGCT	qPCR
		Reverse	ACATCCACCACAAACGCTCC	
prf3b	ENSDARG00000030394	Forward	ACCTTCTCTTGCCTGTTGTG	qPCR
		Reverse	AAAGCTCCGTCCATATAGGG	
gzma	ENSDARG00000090380	Forward	AACACACAGCAGCACAGAGG	qPCR
		Reverse	GAGCCTGAAAGCACTCCAAC	
gzmk	ENSDARG00000028780	Forward	GCAGGAAATACACAGCAGCA	qPCR
		Reverse	AATCCCACATCCTTGTGAGC	
il6	ENSDARG00000102318	Forward	TCAACTTCTCCAGCGTGATG	qPCR
		Reverse	TCTTTCCCTCTTTTCCTCCTG	
tnfa	ENSDARG00000009511	Forward	ACCAGGCCTTTTCTTCAGGT	qPCR
		Reverse	GCATGGCTCATAAGCACTTGTT	
il1b	ENSDARG00000098700	Forward	TTCCCCAAGTGCTGCTTATT	qPCR
		Reverse	AAGTTAAAACCGCTGTGGTCA	
asc	ENSDARG00000040076	Forward	CGGAATCTTTCAAGGAGCAG	qPCR
		Reverse	TGATCGCCCTCAAAATCTCC	
caspa	ENSDARG00000008165	Forward	AAAAGGAGCGGCTCAGAGAA	qPCR
		Reverse	CACCCATAATGGCGTCTCTT	
atg5	ENSDARG00000023396	Forward	AGAGAGGCAGAACCCTACTATC	qPCR
		Reverse	CCTCGTGTTCAAACCACATTTC	
becn1	ENSDARG00000079128	Forward	GATCATGCAATGGTGGCTTTC	qPCR
		Reverse	CCTCCTGTGTCCTCAATCTTT	
gabarapa	ENSDARG00000035557	Forward	GTCTGACCTCACAGTTGGGC	qPCR
		Reverse	TCCTGGTAGAGCAGTCCCAT	
lc3b	ENSDARG00000101127	Forward	CGGTCACAGCATGGTATCTG	qPCR
		Reverse	TCCAAATGTCTCCTGGGAAG	
